# Supplementary material for: Denoising of Aligned Genomic Data
Source: Sci Rep. 2019 Oct 21;9:15067. doi: 10.1038/s41598-019-51418-z (PMC6803637; doi:10.1038/s41598-019-51418-z)
Supplement: Supplementary file 1 — SAMDUDE supplementary data [file 41598_2019_51418_MOESM1_ESM.pdf]

Supplementary data of manuscript "Denoising of Aligned Genomic Data"

Irena Fischer-Hwang, Idoia Ochoa, Tsachy Weissman and Mikel Hernaez

In this supplementary document, we provide details of our implementation. We also discuss the supplementary results that support the main text.

Data

Data sets

The data sets used in this study were obtained from the following sources: ERR262997 (data set 1) with 30×-coverage (`http://www.ebi.ac.uk/ena/data/view/ERA207860`), CEUTrio.HiSeq.WGS (data set 2) with 100×-coverage (`ftp://gsapubftp-anonymous@ftp.broadinstitute.org/bundle/b37/CEUTrio.HiSeq.WGS.b37.NA12878.bam`), and NA12878_V2.5_Robot_2 (data set 3) with 40×-coverage (`https://www.garvan.org.au/research/kinghorn-centre-for-clinical-genomics/clinical-genomics/sequencing-services/sample-data`).

Data coverage

We tested SAMDUDE on three different paired-end WGS datasets of the *H. Sapiens* individual NA12878. The datasets (referred to by run accession number) are: ERR262997 with to 30×-coverage, CEUTrio.HiSeq.WGS with to 100×-coverage, and NA12878_V2.5_Robot_2 with to 40×-coverage, which we refer to these datasets as 1, 2 and 3, respectively in the main text. Denoising was also tested on a dataset with lower (15×) coverage, paired-end WGS dataset ERR174324. The results are shown in Supplementary Table 6, and demonstrate an overall lack of improvement and actual slight negative effect

of SAMDUDE denoising. These results are not entirely surprising, given that SAMDUDE makes significant use of alignment information in order to estimate the noise channel, create counts vectors and to perform denoising, so we expect better denoising performance with higher coverage. Based on these results, we focused our efforts on denoising data with 30× or higher coverage, namely datasets 1, 2 and 3. This tends to be the coverage range of WGS data used in practice, especially for clinical purposes.

SAMDUDE operation

The following subsections discuss specific aspects of SAMDUDE operation used in our experiments.

Choice of k

For all denoising experiments, we used a single-sided context length of $k = 7$ (14 bases total in the double-sided context). This choice is rooted in the theory underlying DUDE, as well as in practical considerations. For discrete universal denoising using DUDE, the optimal single-sided context length k_n depends on both sequence length and alphabet size:

$$k_n = \lceil c \log_{\mathrm{M}} n \rceil$$

with $c < \frac{1}{2}$, noise-free sequence alphabet size M and noise-free sequence length n [1]. Intuitively, the optimal context length maximizes the number of times each context is counted without skewing the context histograms towards a uniform distribution, which occurs when k is either too small or too large. In the genomic sequencing setting, M= 4, and n—interpreted as chromosome length—ranges from 51×10^6 up to 248×10^9 for somatic human chromosomes. For these values, $k_n = 5$ or 6.

Supplementary Table 4 shows the results of denoising extracted SAM files for chromosomes 11 and 20 from dataset 1 using $k = 5$ and 6, and confidence probability threshold $t_{\mathrm{p}} = 0.9$. These values of k resulted in improvements in both S and P, but very little change in F-score. These results seemed to indicate that k was too short, since when k is insufficiently long, information from genomic locations other than the one under consideration may be incorporated, leading to artificially inflated counts in the $\mathbf{m}$ vectors. We hypothesized that denoising performance might be improved by limiting

denoising decisions to reads that map closer to the one under consideration. To test this hypothesis, we tried larger values of k. Intuitively, larger k should ensure that most context counts are taken from the same pileup, while still allowing counts information to be obtained from misaligned or poorly-mapped reads found elsewhere in the SAM file. Results for denoising dataset 1 with $k = 10$ for chromosome 20 are shown in the last line of Supplementary Table 4. Although the best denoising performance was obtained for $k = 10$, a computationally prohibitive amount of memory was required to store the context vectors. Thus, we decided to use $k = 7$.

Choices of t_{m} and t_{p}

For sequence and channel estimation we used a majority threshold of $t_{\mathrm{m}} = 0.9$ for a high-confidence genomic sequence estimate, and also to eliminate the confounding effects due to heterozygous genomic positions which might not have a clear majority base.

The choice of confidence probability threshold t_{p} is a tradeoff. Too high a threshold might prevent the correction of true sequencing errors, while too low a threshold might result in SAMDUDE attempting to denoise where there are no errors. Supplementary Table 5 shows the results of denoising dataset 1 with $t_{\mathrm{m}} = 0.9$ and $t_{\mathrm{p}} = 0.99$, corresponding to a quality score of 20. Compared to the results in Supplementary Table 4, there is less improvement in sensitivity (in fact, worsening of sensitivity for chromosome 20 using $k =$ 6), especially for the GATK filtered variant calls. Additionally, histograms of quality score changes shown in Supplementary Fig. 7 demonstrate that generally SAMDUDE quality score updating tends to shift the quality score distribution towards smaller values. In other words, SAMDUDE denoising decisions tend to decrease the "certainty" of the corrected base. It is unclear how this directly affects variant calling, but intuitively it makes sense to avoid lowering the quality score of bases that were called by the sequencer with high confidence. Finally, a lower threshold of $t_{\mathrm{p}} = 0.9$ has the additional benefit of reducing computational runtime and preventing over-processing of the original data. Based on these results, we chose to use a lower confidence probability threshold of $t_{\mathrm{p}} = 0.9$ for our denoising experiments.

Variations on SAMDUDE

Two "variations" on SAMDUDE were presented in the "Human chromosome denoising with SAMDUDE" subsection of the Results section in the main text: partial denoising and random noise. The results of these variations are summarized in Supplementary Table 1. Supplementary Table 2 lists the total number of bases in the original SAM files, as well as the percent of base changes under each denoiser in the chromosome files for each data set.

Sensitivity vs. precision curves

Supplementary Figs. 1, 2, 3, 4, 5, and 6 show precision as a function of sensitivity for variant call sets filtered by QD. The odd-numbered figures include an additional rightmost point corresponding to the raw variant calls corresponding to those reported in all other tables and figures, while the even-numbered figures omit the rightmost point for ease of visualization.

In all curves, the performance of SAMDUDE is compared with lossy quality score compressors P-Block and R-Block, as well as with Musket and RACER. For ease of visualization, the results of denoising with BFCounter and Lighter are omitted.

Computational details

State-of-the-art denoising software

In this section we provide the commands and parameters used for denoising with Musket, RACER, BFCounter and Lighter.

Musket

Musket was invoked using all default parameters, and the number of threads `t` specified with the `-p` parameter.

Version: 1.1

Website: `http://musket.sourceforge.net/homepage.htm`

Command:

```
$ musket -p t read_1.fastq read_2.fastq -omulti denoised -inorder
```

RACER

RACER was invoked using 3×10^9 as an estimate of the whole human genome length.

Version: 1.0.1

Website: `http://www.csd.uwo.ca/~ilie/RACER/`

Command:

```
$ racer read_1.fastq read_2.fastq 3000000000
```

BFCounter

BFCounter was invoked using estimated sequence lengths of 64×10^6 and 100×10^6 for chromosomes 20 and 11, respectively, and 4×10^9 as an estimate of the whole human genome length.

Version: r181

Website: `https://github.com/lh3/bfc`

Command for denoising chromosome 11 data:

```
$ bfc -s 100m read_1.fastq.gz read_2.fastq.gz
```

Lighter

Lighter was invoked using estimated sequence lengths of 63×10^6 and 135×10^6 for chromosomes 20 and 11, respectively, and k-mer length of 17.

Version: 1.1.1

Website: `https://github.com/mourisl/Lighter/`

Command for denoising chromosome 20 data:

```
$ lighter read_1.fastq read_2.fastq -K 17 63500000
```

Variant calling pipeline

In this subsection we describe the variant calling pipeline.

Preprocessing

Prior to variant calling, all data was preprocessed using the steps recommended by the Broad Institute's Genome Analysis Toolkit (GATK) [2, 3, 4]. They are: conversion of the SAM file to the BAM format, file sorting, duplicate reads marking, group name adding, file indexing and quality score recalibration.

If the denoised file is in FASTQ format, alignment to a reference genome must be performed prior to applying all other preprocessing steps. This was done using the BWA mem alignment program and NCBI build 37 of the human reference [`http://www.ncbi.nlm.nih.gov/assembly/GCF$\_$000001405.13/`] as the reference genome. We included the `-M` option for compatibility with Picard tools (`http://broadinstitute.github.io/picard/`), and used the `-t` option to specify the desired number of threads for computation.

```
$ bwa mem -t num_threads -M ref.fa read_1.fastq read_2.fastq > aln.sam
```

The SAM file was converted to BAM format using SAMtools [?], specifying the number of threads with the `-@` option and inclusion of the SAM header with the `-h` option.

```
$ samtools view -@ num_threads -b -h aln.sam > aln.bam
```

The BAM file was sorted using SAMtools, with a temporary file prefix specified with the `-T` option, and the output file format specified with the `-O` option.

```
$ samtools sort -T ./tmp -@ num_threads -O bam aln.bam > aln.sorted.bam
```

Duplicates were marked using Picard tools, with `M` specifying the file to which metrics calculated during the duplication marking process were written. Note that marking the duplicates is sufficient to exclude them from downstream processes.

```
$ java -jar picard.jar MarkDuplicates I=aln.sorted.bam \
      O=aln.sorted.dedup.bam M=metrics.txt ASSUME_SORTED=true
```

Read group names were then added to the file, with read group parameters specified by `RGID`, `RGLB`, `RGPL`, `RGPU` and `RGSM`.

```
$ java -jar picard.jar AddOrReplaceReadGroups INPUT=aln.sorted.dedup.bam \
      OUTPUT=aln.sorted.dedup.rg.bam RGID=group1 RGLB=lib1 \
      RGPL=illumina RGPU=unit1 RGSM=NA12878
```

Then, the BAM file was indexed.

```
$ java -jar picard.jar BuildBamIndex I=$aln.sorted.dedup.rg.BAM
```

Finally, the quality scores were recalibrated using the GATK Base Quality Score Recalibration workflow [2], and NCBI build 37 of the human reference as the reference genome.

```
$ java -jar GenomeAnalysisTK.jar -nct num_threads -T BaseRecalibrator -R pa
      -I aln.sorted.dedup.rg.bam \
      -knownSites bundle_2.8/dbsnp_138.b37.vcf \
      -knownSites bundle_2.8/Mills_and_1000G_gold_standard.indels.b37.vcf \
      -knownSites bundle_2.8/1000G_phase1.indels.b37.vcf -o bqsr.data
```

```
$ java -jar GenomeAnalysisTK.jar -nct num_threads -T PrintReads -R pathHuma
      -I aln.sorted.dedup.rg.bam -BQSR recal_data -o aln.sorted.dedup.rg.re
```

Variant calling and filtering

We used the GATK Haplotype Caller for variant calling, specifying the target chromosome with the `-L` option.

```
$ java -jar GenomeAnalysisTK.jar -T HaplotypeCaller -R pathHumanReference \
      -I aln.sorted.dedup.rg.recal.bam -L targetRegion \
      --genotyping_mode DISCOVERY -stand_emit_conf 10 -stand_call_conf 30 -
```

Finally, we used the Illumina open source hapolotype comparison tool hap.py (`https://github.com/Illumina/hap.py#happy`) to compare the variant calls of the denoised files with those of the original file. The tool generates comparisons for both raw variant calls as well as variant calls filtered by the GATK Best Practices variant filtering procedure [3]. The hap.py evaluation pipeline was also used to filter extract true and false positive variant call values.

```
$ python hap.py ground_truth.vcf $raw_VCF -f ground_truth.bed -o results -r
$ python rep.py -o results.html -l tsv_file
```

We reported results for both raw variants and variants filtered under the GATK Best Practices-recommended variant filtering process.

Computing requirements

We ran most experiments on a workstation computer with 12 Intel Xeon cores at 3.4 GHz and 32 GB of RAM, running Linux Ubuntu 14.04.4. SAMDUDE

denoising for the chromosome 11 file of dataset 3 was run on a different workstation with 80 Intel Deon cores at 2.2 GHz and 504 GB RAM, running CentOS 7.4.1708. Time and peak computational memory requirements for denoising datasets 1, 2 and 3 using all denoisers are summarized in Supplementary Table 7. In its current manifestation, SAMDUDE generally uses about an order of magnitude more memory than Musket and RACER, and nearly two orders of magnitude more memory than BFCounter and Lighter. This is due to the large number of context histogram vectors that SAMDUDE acquires. SAMDUDE also generally requires about one to two orders of magnitude more runtime than the state-of-the-art denoisers. This result is not surprising, given that SAMDUDE is currently implemented in Python with no parallelization.

Tables and Figures

	data set	SAMDUDE ΔS [%]	SAMDUDE ΔP [%]	SAMDUDE ΔF [%]	Partial denoising ΔS [%]	Partial denoising ΔP [%]	Partial denoising ΔF [%]	Random noise ΔS [%]	Random noise ΔP [%]	Random noise ΔF [%]
raw	1	0.04	0.12	0.08	-0.01	0.19	0.09	-1.39	1.58	0.10
	2	–	0.95	0.47	0.01	0.05	0.03	-0.88	1.00	0.06
	3	–	0.02	0.01	–	-0.01	–	–	0.03	0.01
GATK filtered	1	0.03	0.11	0.07	-0.01	0.12	0.06	-2.29	0.78	-0.76
	2	-0.01	0.75	0.37	0.01	0.02	0.02	-1.39	0.80	-0.29
	3	0.01	–	–	–	0.01	0.01	-0.01	0.02	0.01

Supplementary Table 1: Changes (Δ) in sensitivity (S), precision (P) and F-score (F) under SAMDUDE, SAMDUDE-denoised reads with original quality scores (Partial denoising), and random noise (Random noise) calculated relative to the original file. Positive Δ indicates improvement with respect to the original data, and horizontal lines indicate no change.

chr	data set	n	SAMDUDE [%]	Musket [%]	RACER [%]	BFCounter [%]	Lighter [%]
11	1	5,806,522,969	0.36	0.34	8.41	0.23	0.42
	2	11,960,009,536	1.80	0.53	1.26	0.55	0.62
	3	6,769,559,684	0.07	0.80	1.84	0.89	0.62
20	1	2,538,750,907	0.35	0.30	8.76	0.26	0.42
	2	5,206,460,817	1.80	0.59	1.34	0.63	0.73
	3	3,064,700,879	0.11	1.77	1.99	0.99	0.70

Supplementary Table 2: Total number of bases in the original SAM files (n) compared to the percentage of base changes recommended under the five denoisers.

Quality score bin	Quality score range
1	< 2
2	2–9
3	10–19
4	20–24
5	25–29
6	30–34
7	35–39
8	≥ 40

Supplementary Table 3: Quality score bin labels and ranges.

		Raw variant calls				GATK filtered variant calls			
chr	k	ΔC	ΔS [%]	ΔP [%]	ΔF [%]	ΔC	ΔS [%]	ΔP [%]	ΔF [%]
11	5	26	0.01	0.03	–	50	0.01	0.02	–
	6	-199	0.03	0.17	0.10	-80	0.03	0.11	0.10
20	5	59	0.04	0.07	–	59	0.04	0.06	–
	6	-19	0.01	0.10	–	18	0.01	0.05	–
	10	373	0.21	0.16	0.20	72	0.20	0.12	0.10

Supplementary Table 4: Results of denoising data set ERR262997 with different values of k, with changes (Δ) in T.P. and F.P. calculated relative to the original file. C is the number of additional variants called for each condition relative to the variant call set for the original file. For S, P and F, positive Δ indicates improvement with respect to the original data, and horizontal lines indicate no change.

		Raw variant calls				GATK filtered variant calls			
		ΔC	ΔS	ΔP	ΔF	ΔC	ΔS	ΔP	ΔF
chr	k		[%]	[%]	[%]		[%]	[%]	[%]
11	5	71	0.01	0.03	–	103	0.01	0.02	–
	6	-238	–	0.20	0.10	-112	–	0.12	0.10
20	5	79	0.05	0.05	–	92	0.05	0.03	
	6	-23	-0.01	0.10	–	20	-0.03	0.03	–

Supplementary Table 5: Results of denoising data set ERR262997 with various k with confidence threshold $t_p = 0.99$. C is the number of additional variants called for each condition relative to the variant call set for the original file, and changes (Δ) were calculated relative to the original file. Positive Δ indicates improvement with respect to the original data, and horizontal lines indicate no change.

		Raw variant calls				GATK filtered variant calls			
		ΔC	ΔS	ΔP	ΔF	ΔC	ΔS	ΔP	ΔF
chr	k		[%]	[%]	[%]		[%]	[%]	[%]
11	5	-13	0.01	–	–	-7	0.01	–	–
	6	-209	–	0.01	–	-220	-0.02	0.01	–
	7	-451	-0.03	0.03	–	-382	-0.03	–	–
20	5	18	–	-0.01	–	39	0.01	–	–
	6	-111	-0.03	0.01	–	-103	-0.04	–	–
	7	-153	-0.02	–	–	-117	-0.03	–	–
	10	235	0.05	-0.04	–	189	0.03	-0.02	–

Supplementary Table 6: Results of denoising low coverage data set ERR174324 with confidence threshold $t_p = 0.9$. C is the number of additional variants called for each condition relative to the variant call set for the original file, and changes (Δ) were calculated relative to the original file. Positive Δ indicates improvement with respect to the original data, and horizontal lines indicate no change.

		SAMDUDE		Musket		RACER		BFCounter		Lighter	
chr	data set	Memory [GB]	Time [s/MB]	Memory [GB]	Time [s/MB]	Memory [GB]	Time [s/MB]	Memory [GB]	Time [s/MB]	Memory [GB]	Time [s/MB]
11	1	27.45	0.89	2.76	0.42	7.66	0.05	5.18	0.55	0.66	0.17
	2	31.79	3.77	6.53	0.56	16.07	0.08	5.22	0.36	0.66	0.12
	3	77.57	4.37	5.52	0.76	11.05	0.06	5.16	0.43	0.66	0.14
20	1	31.90	6.12	1.06	0.60	3.50	0.06	2.94	0.37	0.33	0.17
	2	31.74	2.58	2.14	0.72	7.35	0.05	2.94	0.35	0.33	0.14
	3	31.86	4.37	1.73	0.76	5.08	0.06	2.85	0.4	0.33	0.11

Supplementary Table 7: Time and peak memory requirements for denoising individual chromosome files from data sets 1, 2 and 3 using the five denoisers. RACER, BFCounter and Lighter requirements are averaged between the paired-end files.

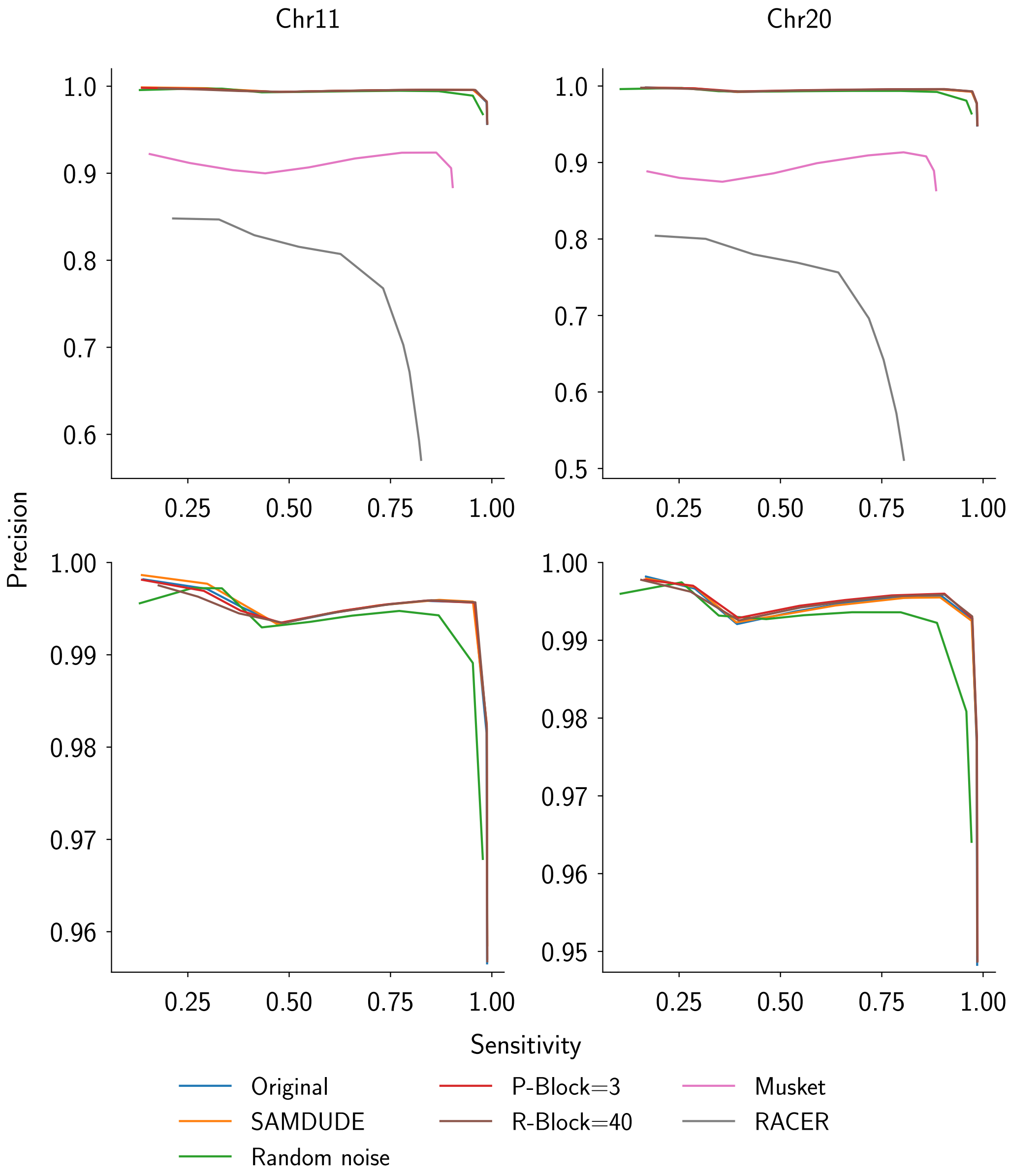

Supplementary Figure 1: Sensitivity vs. precision curves for the data set 1 variant call set filtered at 10th percentiles, starting with no filtering. The top row shows results for all files, and the bottom row omits results for Musket- and RACER-denoised files for closer comparison.

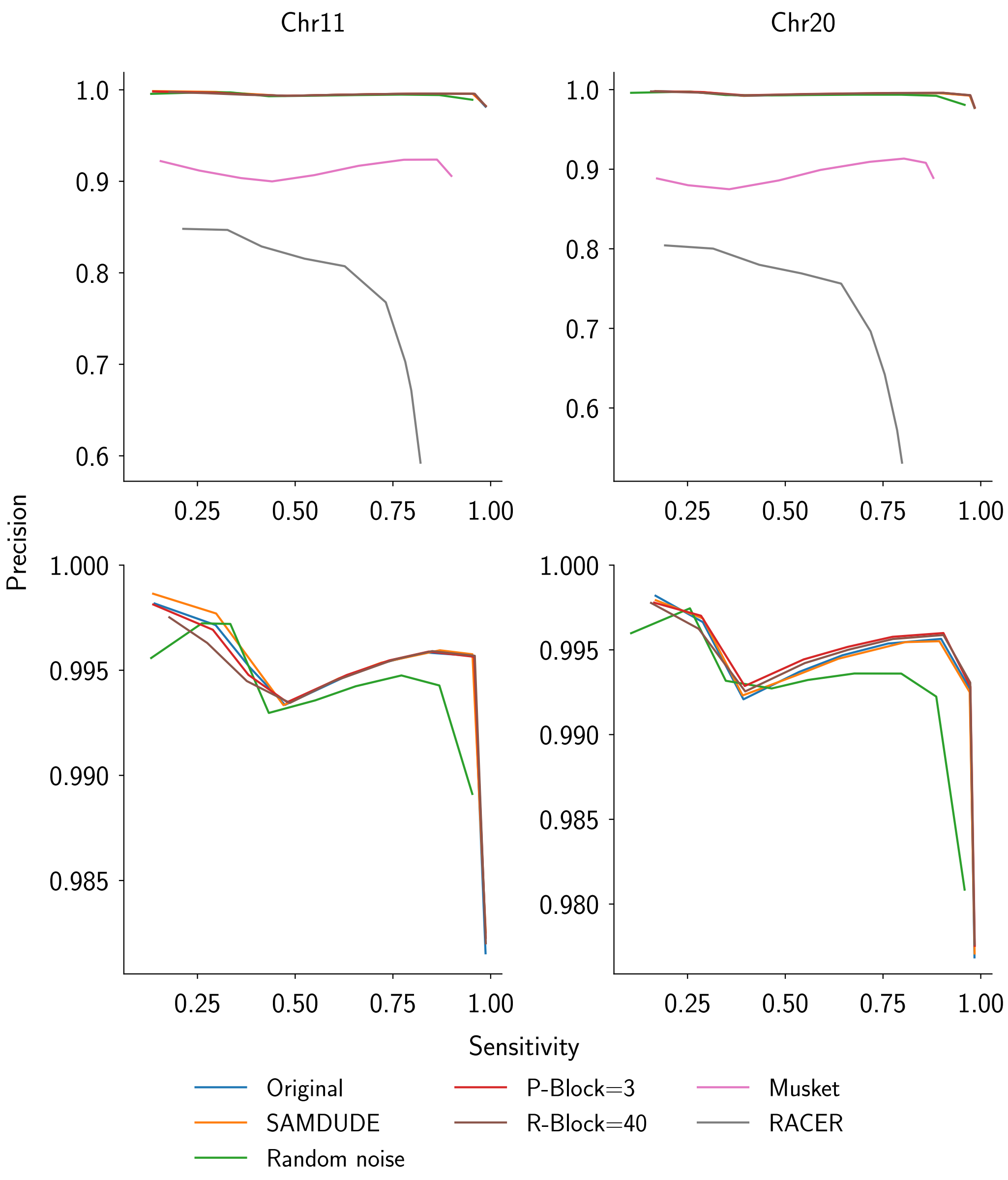

Supplementary Figure 2: The same sensitivity vs. precision curves as in Supplementary Fig. 1, but with the rightmost point in each subplot removed for ease of visualization.

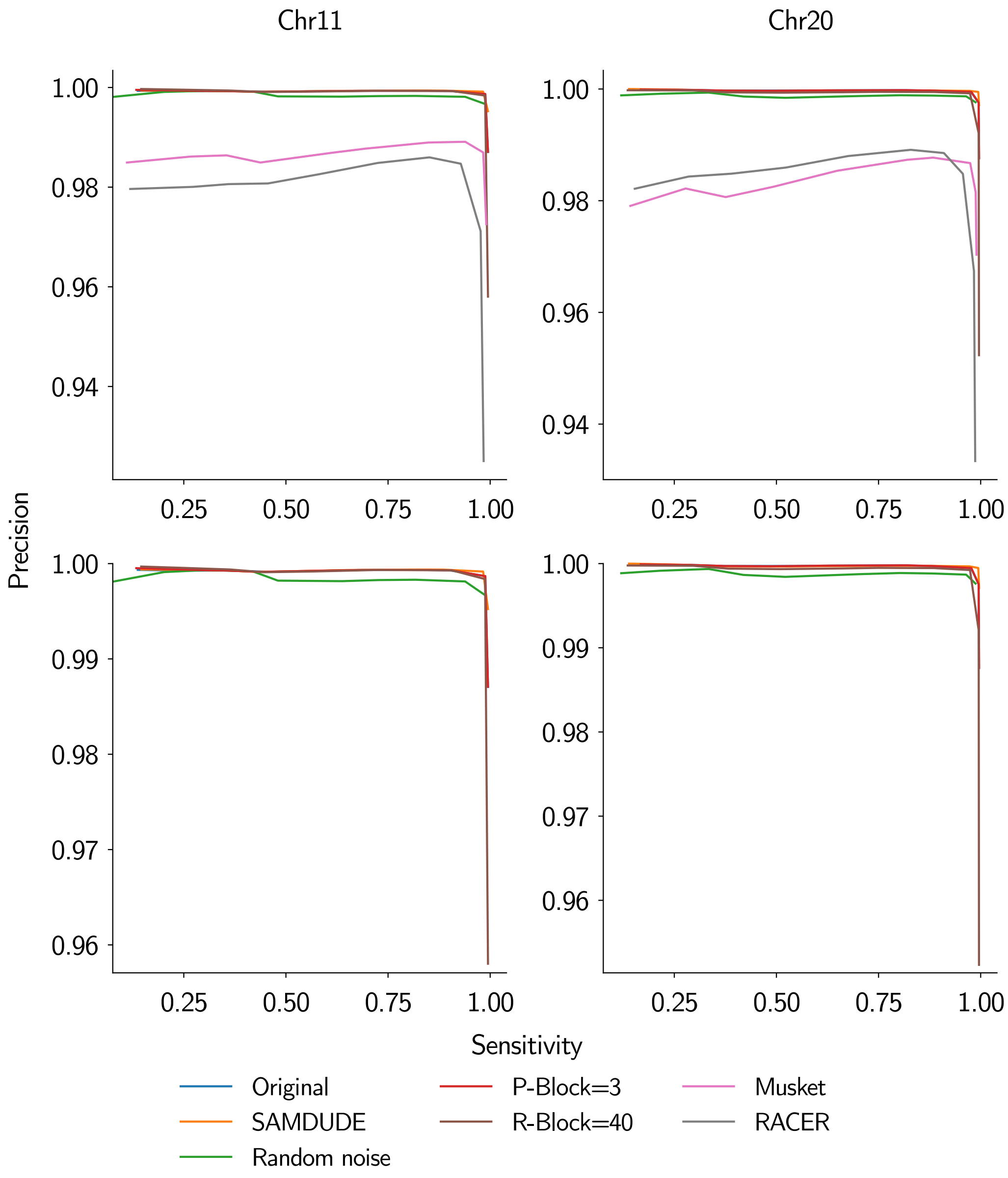

Supplementary Figure 3: Sensitivity vs. precision curves for the data set 2 variant call set filtered at 10th percentiles, starting with no filtering. The top row shows results for all files, and the bottom row omits results for Musket- and RACER-denoised files for closer comparison.

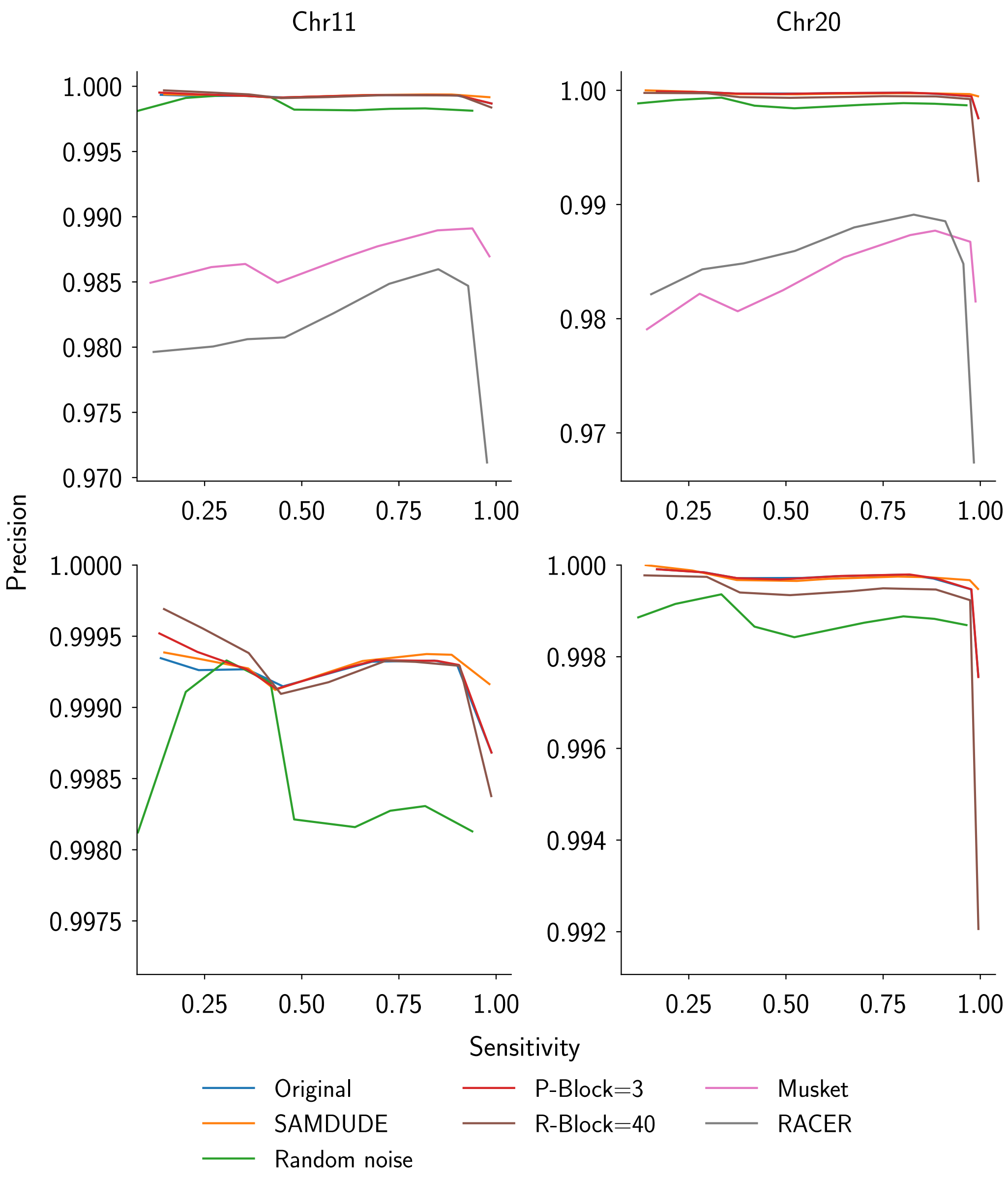

Supplementary Figure 4: The same sensitivity vs. precision curves as in Supplementary Fig. 3, but with the rightmost point in each subplot removed for ease of visualization.

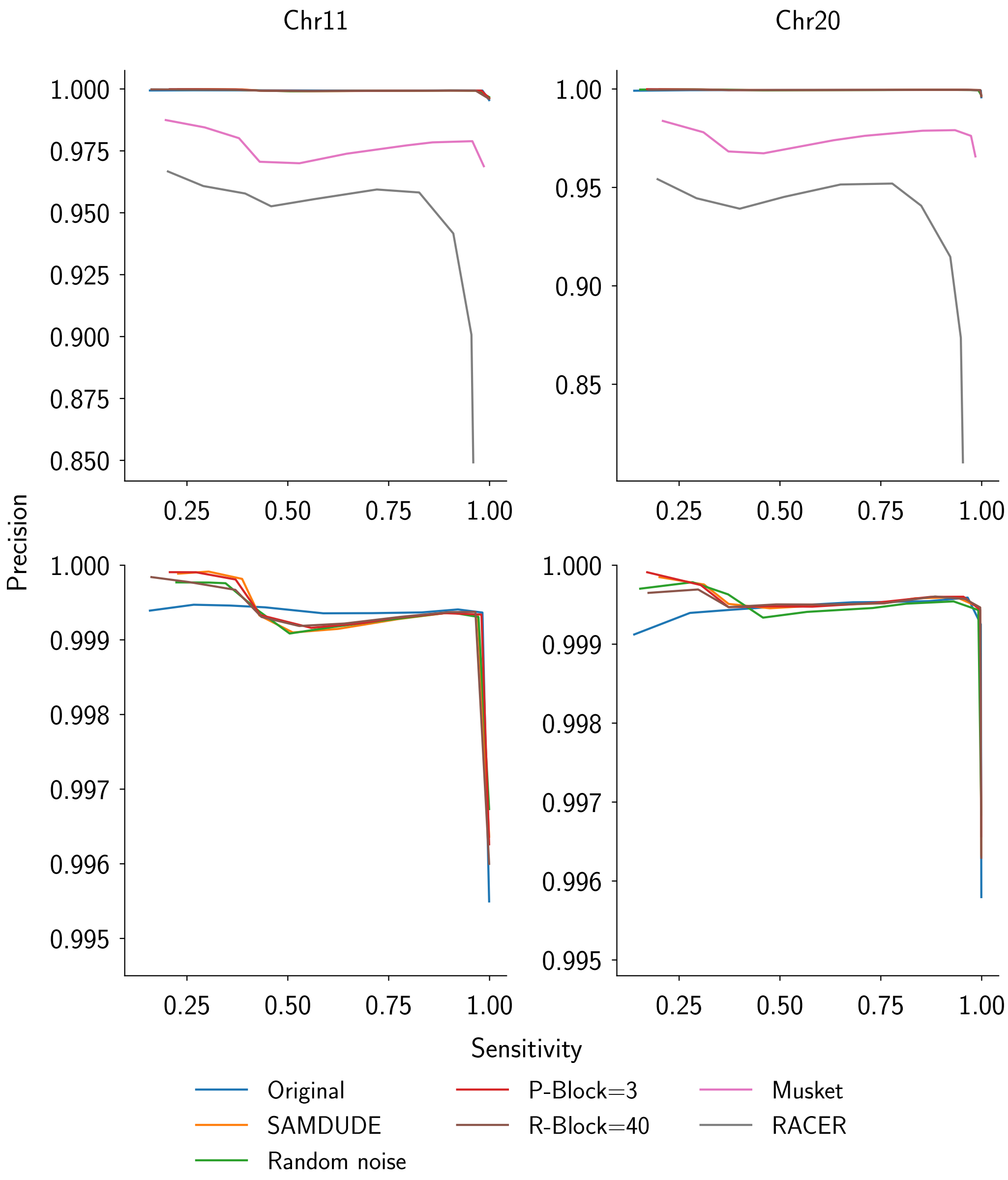

Supplementary Figure 5: Sensitivity vs. precision curves for the data set 3 variant call set filtered at 10th percentiles, starting with no filtering. The top row shows results for all files, and the bottom row omits results for Musket- and RACER-denoised files for closer comparison.

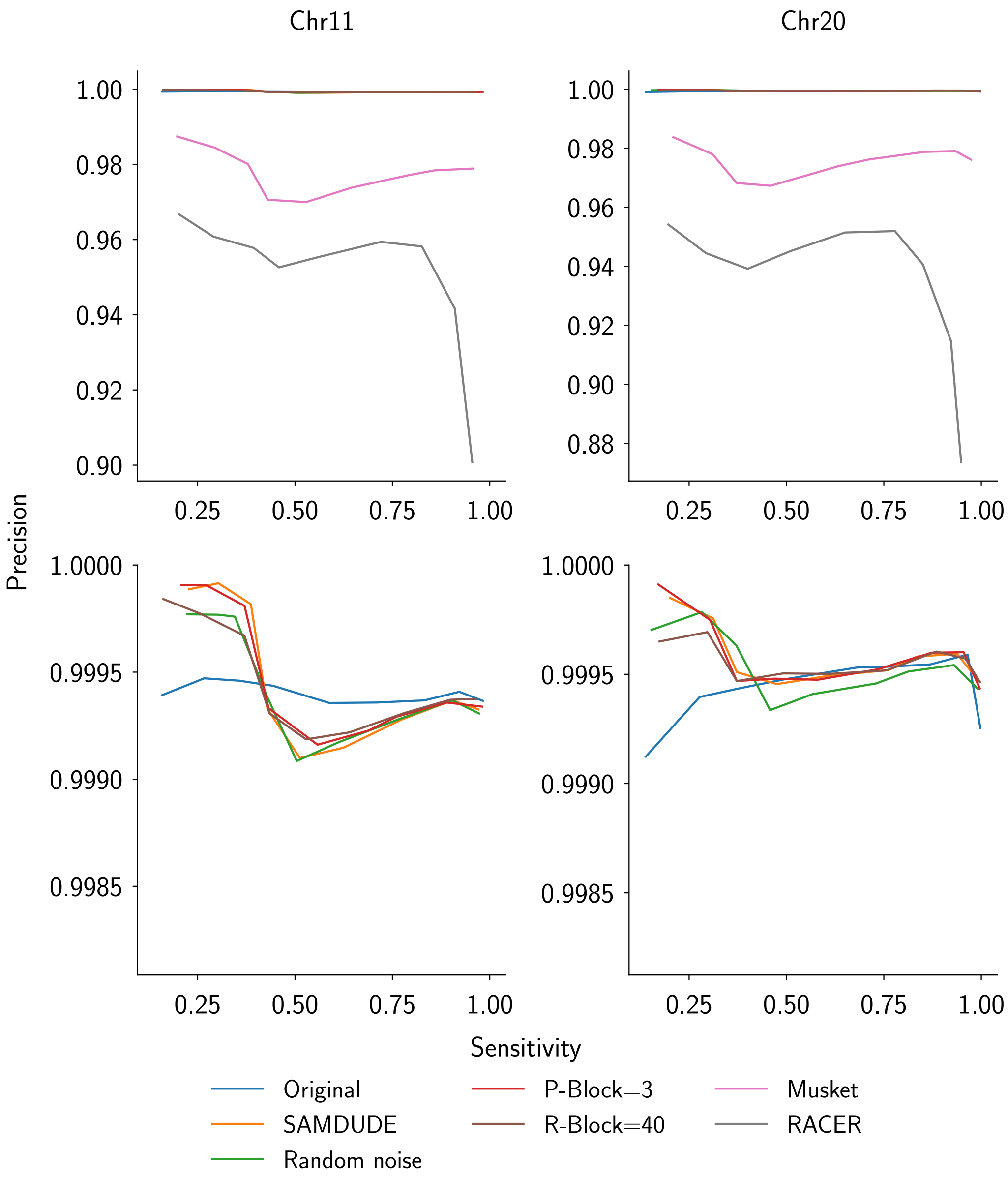

Supplementary Figure 6: The same sensitivity vs. precision curves as in Supplementary Fig. 5, but with the rightmost point in each subplot removed for ease of visualization.

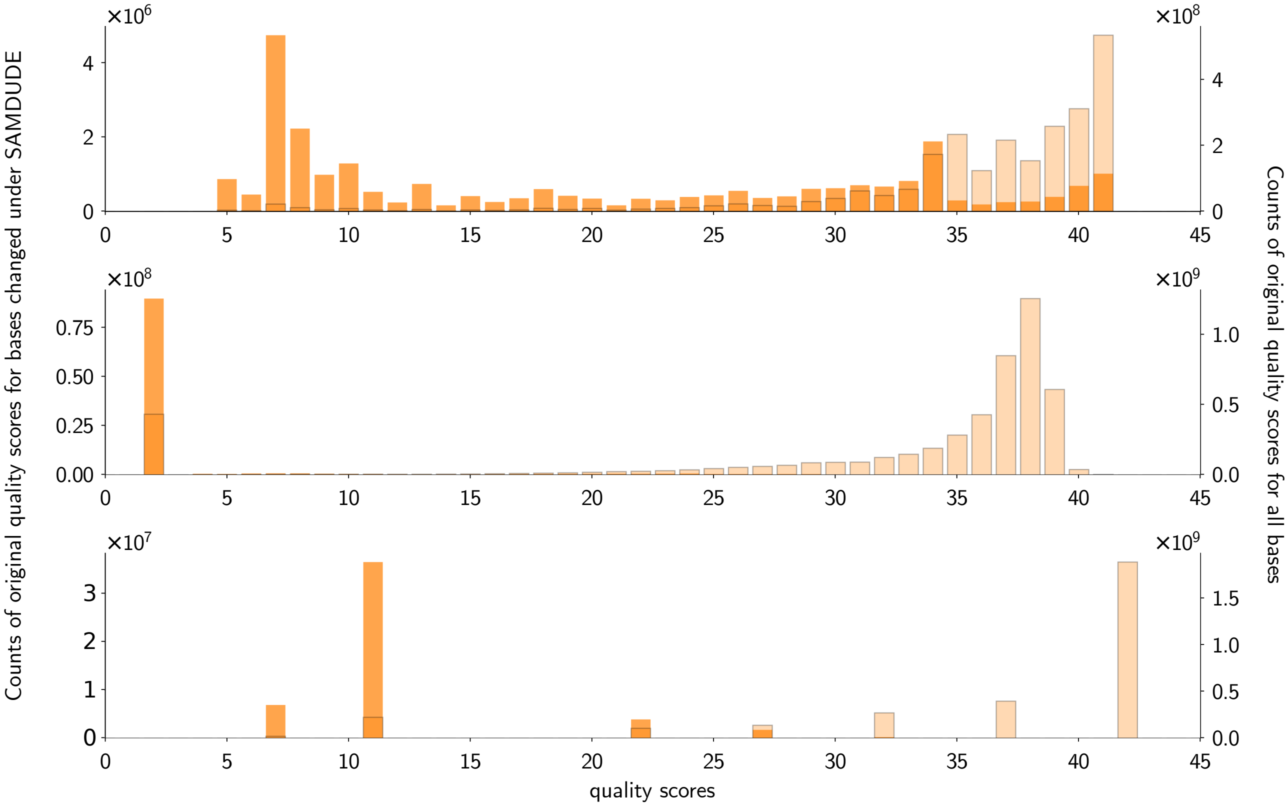

Supplementary Figure 7: From top to bottom: quality score histograms for chromosome 20 reads taken from datasets 1, 2 and 3. Histograms summarize all original quality scores (light orange and outlined, right-hand axis) and scores only of bases changed under the SAMDUDE denoising rule using $k = 7$, $t_{\mathrm{m}} = 0.9$ and $t_{\mathrm{p}} = 0.9$ (dark orange, left-hand axis).

References

[1] T. Weissman, E. Ordentlich, G. Seroussi, S. Verdú, and M. J. Weinberger, "Universal discrete denoising: Known channel," *IEEE Transactions on Information Theory*, vol. 51, no. 1, pp. 5–28, 2005.

[2] A. McKenna, M. Hanna, E. Banks, A. Sivachenko, K. Cibulskis, A. Kernytsky, K. Garimella, D. Altshuler, S. Gabriel, M. Daly *et al.*, "The genome analysis toolkit: a mapreduce framework for analyzing next-generation dna sequencing data," *Genome research*, vol. 20, no. 9, pp. 1297–1303, 2010.

[3] M. A. DePristo, E. Banks, R. Poplin, K. V. Garimella, J. R. Maguire, C. Hartl, A. A. Philippakis, G. Del Angel, M. A. Rivas, M. Hanna *et al.*, "A framework for variation discovery and genotyping using next-generation dna sequencing data," *Nature genetics*, vol. 43, no. 5, pp. 491–498, 2011.

[4] G. A. Van der Auwera, M. O. Carneiro, C. Hartl, R. Poplin, G. del Angel, A. Levy-Moonshine, T. Jordan, K. Shakir, D. Roazen, J. Thibault *et al.*, "From fastq data to high-confidence variant calls: the genome analysis toolkit best practices pipeline," *Current protocols in bioinformatics*, pp. 11–10, 2013.
